# Sporadic Fusobacterium Bacteremia as an Atypical Cause of Acute Hepatitis in a Young Caucasian Woman

**DOI:** 10.7759/cureus.10590

**Published:** 2020-09-22

**Authors:** Nishat Ahmed, Muhammad Hafiz Kamarul Bahrin, Adnan Agha, Anand Deshmukh

**Affiliations:** 1 Acute Medicine, Queen's Hospital Burton, Burton-on-Trent, GBR; 2 Internal Medicine, Queen's Hospital Burton, Burton-on-Trent, GBR; 3 Acute Medicine/Endocrine and Diabetes, Queen's Hospital Burton, Burton-on-Trent, GBR; 4 Microbiology, University Hospital Coventry and Warwickshire, Coventry, GBR

**Keywords:** acute hepatitis, fusobacterium

## Abstract

Fusobacterium species are gram-negative anaerobic non-spore-forming bacteria, which colonize mucous membranes in humans. Over the recent decade, the significance of these organisms has been increasingly recognized. We describe a rare case of acute hepatitis, which was found to be likely due to Fusobacterium nucleatum, grown on blood culture. In our case, the hepatitis caused by this microorganism resolved completely without any long-term sequelae to the liver, through conservative management namely intravenous antibiotics and supportive therapy only. This case highlights that early detection and prompt treatment in a case of acute hepatitis resulted in a good outcome. In addition, this case also illustrates that the differential diagnosis can be varied in cases of acute hepatitis.

## Introduction

Fusobacterium species are commensal to the oral cavity of humans and animals. They rarely cause problems in a healthy human being. In a retrospective study, based on the data obtained from a regional microbiology laboratory serving a population of 1.3 million people, over 11 years, it was found that Fusobacterium species, bacteremia in particular, is exceptionally rare with an annual incidence of only 0.55 cases/100,000 population [1]. The two commonly reported species of Fusobacterium are Fusobacterium nucleatum and Fusobacterium necrophorum, of which the latter is rarer. We report a case of acute hepatitis, associated with Fusobacterium septicemia. Fusobacterium can sometimes be associated with upper respiratory tract infections, which may spread to the internal jugular vein and cause thrombophlebitis. This is known as Lemierre’s disease. The mainstay of treatment is surgical drainage of any resulting secondary abscess and use of antibiotics [2]. The commonly used antibiotics to treat Fusobacterium infections are penicillins and metronidazole. In our patient, she was treated with piperacillin-tazobactam and metronidazole.

## Case presentation

A 27-year-old female patient presented with abdominal pain, nausea, and vomiting of one-week duration. She described an unremitting lower abdominal pain with pain intensity of 7/10, located mainly in the suprapubic region, and was colicky in nature. She had a history of treated chlamydia infection two years ago. There was no history of any chronic illness, and her regular medications only included oral contraceptive pills. She worked in the food industry and did not go to work for the preceding 12 weeks due to the Covid-19 outbreak and the introduction of lockdown measures.

On examination, she appeared jaundiced and demonstrated grade 2 hepatic encephalopathy, as per the West Haven Score, as she was lethargic and minimally disoriented in time and place. She was very tender over the suprapubic region as well as moderately tender all over her abdomen without any rebound tenderness or rigidity. There was no ascites or stigmata of chronic liver disease. Her observation parameters on admission were stable with a temperature of 36.4-degree Celsius, heart rate of 79 beats per minute, respiratory rate of 16 breaths per minute, blood pressure of 91/59 mmHg, and oxygen saturation of 100% on room air. Interestingly, she was never pyrexial throughout her admission. Speculum examination revealed brownish vaginal discharge and tenderness around the adnexa. Cervical swab samples were sent to screen for gonorrhea and chlamydia, along with high vaginal swab for routine culture and sensitivity investigation. 

Admission bloods are shown in Table 1.

**Table 1:** Admission investigation results for full blood picture and liver function tests

Laboratory investigations	Result	Normal range
White blood cell	8.0	4.5-11 x 10^9 ^cells/L
Platelets	68	150-350 x 10^9^/L
Hb	146	140-170 g/dL
Phosphate	0.26	0.8-1.4 mmol/L
International normalized ratio (INR)	2.0	0.9-1.2
Ferritin	3,394	12-200 micrograms/L
Total bilirubin	47	3-17 micromol/L
Alanine aminotransferase (ALT)	8,740	5-35 IU/L
Alkaline phosphatase	112	30-150 IU/L
C-reactive protein	31	<10 mg/L
Albumin	46	35-50 g/L
Paracetamol level	2	

Lactate level on the venous blood gas was initially 3.5 mmol/L and following intravenous (IV) fluid resuscitation, this improved to 1.0 mmol/L.

**Figure 1:**
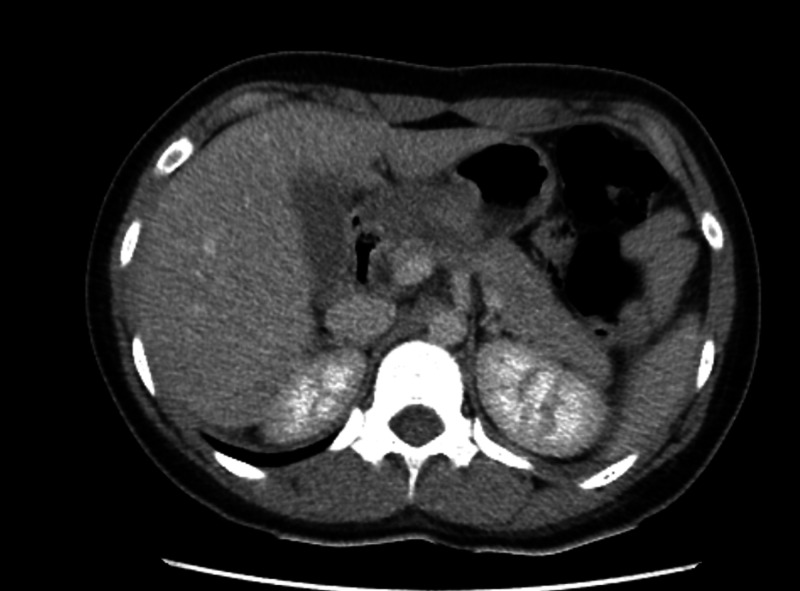
Normal CT image of the liver despite grossly abnormal liver function tests

She underwent CT imaging of the abdomen and pelvis which was later reported as normal, as it did not reveal any convincing etiology for her abdominal pain (Figure 1).

Further initial management included maintenance IV fluids, IV vitamin K 10 milligrams once a day for three days, IV N-acetylcysteine (NAC) infusion 6,000 milligrams over 16 hours, IV piperacillin-tazobactam 4.5 grams thrice daily, alongside oral doxycycline 100 milligrams twice daily, as there was a query of co-existing acute pelvic inflammatory disease (PID). 

Early microbiology review raised a possibility of a Fitz-Hugh-Curtis syndrome given her previous sexually transmitted disease (STD) infection. However, this was invalidated as her screening tests for chlamydia and gonorrhea infection were subsequently negative. 

On day 3 of admission, her blood cultures from the admission grew gram-negative bacteria in one of two of blood culture bottles and subsequent microbiobiolgy advice was to continue the same antibiotics. Her urine culture was reported as negative. The advice from the microbiology team was to continue piperacillin-tazobactam and doxycycline. The microbiology team also requested that other intra-abdominal sources of infection, such as spontaneous bacterial peritonitis (SBP) or the upper urinary tract infection (UTI), be investigated and considered in the differential diagnoses. However, these were deemed unlikely as the patient presented with no ascites and symptoms of UTI were absent, in addition to the negative urine culture result.

She was tested for viral and autoimmune hepatitis, which were all reported as negative. Cytomegalovirus (CMV) and Epstein-Barr virus (EBV) IgG assays were positive, but IgM assays were negative suggesting no acute viral infection from these agents. Covid-19 swab tests were negative on two separate occasions. Fusobacterium nucleatum was later isolated in one of the two blood culture bottles with sensitivity to metronidazole. High vaginal swab result came back as heavy growth of mixed coliforms.

The patient completed two-week course of IV antibiotics, consisting of tazobactam-piperacillin 4.5 grams thrice daily, in the first instance, which was subsequently changed to amoxicillin-clavulanate 1.2 grams thrice daily IV and metronidazole 500 milligrams IV every eight hours. Her liver function tests (LFTs) improved gradually following the course of antibiotics as shown in Table 2, her abdominal pain subsided, and she was discharged home. 

**Table 2:** Serial liver function tests

	17/6/2020	23/6/2020	27/7/2020	
Laboratory investigations	Result	Result	Result	Normal range
International normalized ratio (INR)	2.0	1.3	1.0	0.9-1.2
Total bilirubin	45	60	30	3-17 micromol/L
Alanine aminotransferase (ALT)	7,697	1,623	28	5-35 IU/L
Alkaline phosphatase	84	93	81	30-150 IU/L
Albumin	37	35	52	35-50 g/L

## Discussion

Fusobacterium species colonize the mucous membranes of animals and humans and occasionally cause infections of oral cavity and head and neck. Fusobacterium infections are relatively rare in the UK [2]. Hence, it is also referred to as the forgotten disease [3,4]. It has been reported that Fusobacterium nucleatum is widely present in pregnancy complications, including premature birth and stillbirth [5], as well as intrauterine infections, including neonatal sepsis [6]. Other literature search of Fusobacterium-related pathologies through Medline revealed several case series, describing this microorganism as a common cause of Lemierre's disease. This is a condition associated with anaerobic bacterial infection, starting as sore throat, progressing to severe systemic illness and formation of septic thrombophlebitis. Metastasis to distant organs causing multi-organ failures may occur. Gastrointestinal involvement of Fusobacterium is often manifested as abscess collection or portal vein thrombosis [7]. In some other cases, there has been an interest in the association of this bacterium with the development of colon cancer [8]. 

In this case, the authors speculate that the source of her infection originated from the genital tract mucosa. The history of STD and chronic vaginal discharge may have predisposed to the invasion of deeper tissues with this bacterium and subsequent hematogenous spread. Fusobacterium nucleatum can invade the epithelial cells [9]. Adherence and invasion are essential mechanisms for colonization, dissemination, evasion of host defense, and induction of host responses [10]. This is the viewpoint of the authors for the possible source of infection in this particular case; however, further studies will be required to show a link between female genital infection and bacteremia with this organism.

This case highlights the rare incidence of acute hepatitis as a complication of Fusobacterium nucleatum bacteremia, which was successfully treated with conservative management only. Initially, potential diagnosis of Fitz-Hugh-Curtis syndrome was raised. However, in Fitz-Hugh-Curtis syndrome, the authors understand that the involvement of the liver parenchyma is minimal to none and it mainly affects the hepatic capsule [11], causing hepatic-peritoneal adhesion. As a result, in Fitz-Hugh-Curtis syndrome, LFTs would typically be normal or minimally elevated [12]. Our case, on the other hand, presented with grossly deranged LFTs, indicating an underlying liver parenchymal involvement. From this case, the authors would also like to highlight the importance of early initiation of antibiotics and fluids in the management of Fusobacterium-related hepatitis, with close observation for development of any complications.

## Conclusions

Acute hepatitis from bacterial infections such as Fusobacterium can rarely happen, and the authors wish to raise the awareness of this condition. It is also important to consider sending blood cultures in a patient with abdominal pain and deranged LFTs to diagnose this condition and initiate early antibiotic treatment. To the best of the authors' knowledge, this is a novel case where Fusobacterium bacteremia was associated with acute hepatitis picture without abscess formation.
